# Validated LC-MS/MS Method for the Quantification of Ponatinib in Plasma: Application to Metabolic Stability

**DOI:** 10.1371/journal.pone.0164967

**Published:** 2016-10-20

**Authors:** Adnan A. Kadi, Hany W. Darwish, Mohamed W. Attwa, Sawsan M. Amer

**Affiliations:** 1 Department of Pharmaceutical Chemistry, College of Pharmacy, King Saud University, P.O. Box 2457, Riyadh, 11451, Kingdom of Saudi Arabia; 2 Analytical Chemistry Department, Faculty of Pharmacy, Cairo University, Kasr El-Aini St., Cairo, 11562, Egypt; Purdue University, UNITED STATES

## Abstract

In the current work, a rapid, specific, sensitive and validated liquid chromatography tandem mass-spectrometric method was developed for the quantification of ponatinib (PNT) in human plasma and rat liver microsomes (RLMs) with its application to metabolic stability. Chromatographic separation of PNT and vandetanib (IS) were accomplished on Agilent eclipse plus C18 analytical column (50 mm × 2.1 mm, 1.8 μm particle size) maintained at 21±2°C. Flow rate was 0.25 mLmin^-1^ with run time of 4 min. Mobile phase consisted of solvent A (10 mM ammonium formate, *p*H adjusted to 4.1 with formic acid) and solvent B (acetonitrile). Ions were generated by electrospray (ESI) and multiple reaction monitoring (MRM) was used as basis for quantification. The results revealed a linear calibration curve in the range of 5–400 ngmL^-1^ (r^2^ ≥ 0.9998) with lower limit of quantification (*LOQ*) and lower limit of detection (*LOD*) of 4.66 and 1.53 ngmL^-1^ in plasma, 4.19 and 1.38 ngmL^-1^ in RLMs. The intra- and inter-day precision and accuracy in plasma ranged from1.06 to 2.54% and -1.48 to -0.17, respectively. Whereas in RLMs ranged from 0.97 to 2.31% and -1.65 to -0.3%. The developed procedure was applied for quantification of PNT in human plasma and RLMs for study metabolic stability of PNT. PNT disappeared rapidly in the 1st 10 minutes of RLM incubation and the disappearance plateaued out for the rest of the incubation. *In vitro* half-life (t_1/2_) was 6.26 min and intrinsic clearance (CL_in_) was 15.182± 0.477.

## Introduction

**Fig 1:**
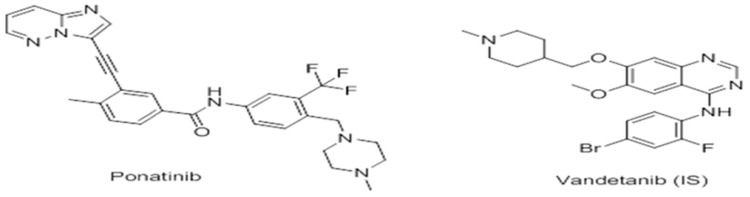
Chemical structure of ponatinib (PNT) and Vandetanib (IS).

Ponatinib (PNT; Fig 1), marketed as (Iclusig^™^ tablets), is a drug taken orally for the management of some types of tumers including chronic Philadelphia chromosome—positive acute lymphoblastic leukemia (Ph^+^ ALL) and myeloid leukemia (CML) [1].

Chimeric breakpoint cluster region-abelson (BCR-ABL) protein (encoded by *BCR-ABL* oncogene) is responsible for the activity of ABL tyrosine kinase which is considered the main reason of chronic myeloid leukaemia (CML) [2]. Some CML cases have recently reported in the literature resistant to common TKIs (e.g. dasatinib and imatinib). However, PNT was found to be still effective against such resistance [3].

Upon literature review, there was only one reported spectrofluorimetric method to quantify PNT in spiked plasma and urine [4]. In this method, the recovery % of PNT in plasma was around 85%. The proposed procedure was fast, sensitive, specific and reproducible. Moreover, it is considered the first validated LC-MS/MS for assaying PNT. In this study, validated and reliable LCMS-MS assay for the analysis of PNT in plasma and RLMs is described. And to the best of the authors knowledge, the method is the first LC-MS based method of its kind.

The current procedure is utilized for estimating the metabolic stability of PNT in RLMs by measuring the rate of its disappearance in RLMs incubation. *In vitro* half-life (t_1/2_) and intrinsic clearance (CL_int_) were utilized for expressing metabolic stability. Based on these two parameters, secondary pharmacokinetic parameters, such as hepatic clearance (CL_H_), bioavailability and *in vivo* t_1/2_ can be calculated which is crucial for establishing *in vivo*—*in vitro* correlation for proper metabolic stability study. If a test compound is rapidly metabolized, its bioavailability *in vivo* will probably be low [5].

## Experimental

### Chemicals and reagents

PNT and vandetanib were purchased from LC Laboratories (Woburn, MA, USA). Ammonium formate, HPLC-grade acetonitrile (ACN) and formic acid (HCOOH) were purchased from Sigma-Aldrich (West Chester, PA, USA). Ultrapure water was obtained from Milli-Q plus purification system, Millipore, Waters (Millipore, Bedford, MA, USA). Human plasma was supplied by King Khaled University Hospital (Riyadh, KSA), after approved permissions were obtained from donners and kept at -70°C till usage. RLMs were prepared in-house following a published method using Sprague Dowely rats [6].

### Chromatographic conditions

An Agilent HPLC-MS/MS (6410 QqQ) was used for chromatographic separation for the PNT (analyte) and vandetanib (IS). HPLC (Agilent 1200 series) system consisting of binary pump (G1311A), degasser (G1322A), Autosampler (G1367B) and thermostatted column compartment (G1316) and an Agilent 6410 QqQ LC/MS (Agilent Technologies, Palo Alto, CA, USA) with an electrospray ionization (ESI) interface. The chromatographic separation was performed on Agilent eclipse plus C_18_ analytical column (50 mm × 2.1 mm, 1.8 μm particle size) (Agilent Technologies, Palo Alto, CA, USA). Column temperature was kept constant at 21±2°C. All chromatographic conditions were optimized to achieve the best separation in a very short time. Flow rate was 0.25 mL min^-1^. Mobile phase consisted of solvent A which is 10 mM ammonium formate (*p*H: 4.1 by addition of formic acid) and solvent B which is acetonitrile. Sample injection volume was 5 μL with a total run time of 4 min. Optimization of mass spectrometer was done for PNT and vandetanib. Detection was performed on a QqQ mass detector, operated with an ESI positive ionization mode. Nitrogen was used as drying gas at a flow rate of 11 L/min and high purity nitrogen as collision gas at a pressure of 50 psi. Source temperature and capillary voltage were set at 350°C and 4000 V respectively. Mass Hunter software (Agilent Technologies, Palo Alto, CA, USA) was used to control the instruments and data acquisition. Assaying of PNT was carried out utilizing the mode of multiple reaction monitoring (MRM) for the transitions 533→433 and 533→260 for PNT and 475→112 for vandetanib (IS). Fragmentor voltage was set to 140 and 145 V with collision energy of 16, 15 for PNT and 145 V with collision energy 15 for vandetanib.

### Preparation of standard solutions

PNT standard solution was prepared in DMSO to produce a final concentration of 1.0 mgmL^-1^. One mL of stock solution was diluted to 10 mL with mobile phase to give a 100 μgmL^-1^ (working standard 1) then 1 mL of this solution was diluted to 10 mL with mobile phase to produce 10 μgmL^-1^ (working standard 2). For vandetanib (IS) stock solution, reference powder was solubilized in DMSO to produce a concentration of 0.1 mgmL^-1^. Two hundred μL of this stock solution were diluted to 10 mL with mobile phase producing a working solution of 2 μgmL^-1^.

### Preparation of rat liver microsomes (RLMs) matrix

Four Sprague-dawley rats are supplied by animal care center belongs to the faculty of pharmacy, King Saud University. The animal experimental protocol was approved by the Institutional Review Board at King Saud University. Peritoneal cavity incision was carried out after cervical dislocation of the rats to take liver. The rat livers were then transferred to a clean backer and weighed. Buffer (pH 7.4) containing 0.04M KH_2_PO_4_/NaH_2_PO_4_, 0.25M sucrose and 0.15M KCl (KCl/sucrose buffer) was added to the rat liver (1/4 W/V). Liver pieces were then homogenized (1/4 W/V) using OMNI homogenizer (Omni International, Kennesaw, GA, USA). The liver homogenate was centrifuged at 9000 g for 25 min at 4°C. The supernatant was collected and then centrifuged at a 100000 g for 65 min followed by supernatant removal and pellets were suspended in KCl/sucrose buffer. Microsomal suspension was freezed at -76°C and Lowry method [7] was adopted for assaying its protein content. Evaluation of CYP450 activity was assessed by ability of RLMs to bioactivate phenytoin to *p*-hydroxyphenytoin [8].

### Sample preparation procedure and construction of the calibration curve

Appropriate volumes of PNT working standard solution (10 μgmL^-1^) were diluted in RLMs matrix and human plasma to prepare two sets of eleven concentrations: 5, 10, 20 (low quality control; LQC), 30, 40, 50, 100, 150 (medium quality control; MQC), 200, 300 (high quality control; HQC) and 400 ngmL^-1^. One mL of 0.1 M NaOH/glycine buffer (pH∼9) was added and samples for were shaken for mixing for 15 seconds. Two mL of ACN was added for protein precipitation. Precipitated proteins are removed by centrifugation at 14000 rpm for 12 min at 4°C. Supernatants was removed and filtered through a Millex-GP, 0.22 μm syringe filter (Millipore, Billerica, MA, USA). IS (50 μL) was added to 1 mL of the filtered standards and then loaded in the auto-sampler tray and volumes of 5 μL were injected into LC-MS/MS. Blank was prepared by processing of free drug RLMs matrix or human plasma with similar procedure using mobile phase instead, then tested to ascertain the absence of any endogenous interference with PNT and IS. Two calibration curves (5, 10, 20, 30, 40, 50, 100, 150, 200, 300 and 400 ngmL^-1^) were generated for spiked human plasma samples and spiked RLMs samples by drawing the ratio of peak area of PNT to that of IS (*y* axis) against PNT nominal concentrations (*x* axis). Analysis of each concentration of PNT was achieved in triplicates. Linear regression was expressed using various parameters such as slope, intercept, and r^2^ values. The concentrations of PNT in various spiked RLMs samples or spiked plasma samples were computed by substitution of PNT and IS peak area ratios in the linear regression equation.

### Method validation

General regulations stated by International Conference on Harmonisation (ICH) [9, 10] and Food and Drug Administration (FDA) guidelines for analytical procedures and methods validation [11] were followed for validation purposes.

#### Specificity

For investigation of the method specificity, six blank RLMs and human plasma matrix samples were exposed to the same proposed extraction procedures. Then these samples were assayed for any interference peaks at retention time of PNT or IS and matching the chromatogram with PNT and IS spiked RLMs and plasma matrix samples. For minimizing carryover effects in the mass detector, MRM mode was used.

#### Linearity and sensitivity

Linearity and sensitivity of the suggested procedure were assessed utilizing six different calibration curves. Each calibration curve was generated by plotting the peak area ratio of PNT to the vandetanib (IS) as *y* axis versus the nominal concentrations of PNT as *x* axis. Calibration curves for PNT in spiked RLMs and plasma matrices were prepared in the same day of analysis at 11 different concentrations (in the range of 5 to 400 ngmL^-1^). Statistical least square method was applied for the analysis of the resulting data. Limit of detection (*LOD*) and limit of quantitation (*LOQ*) were calculated according to the ICH guidelines [9], by applying the following formula.

LOQ OR LOD=mSn

Where *m* equals 3.3 for *LOD* and 10 for *LOQ*, *S* is the standard deviation of the intercept, and *n* is the slope.

#### Precision and accuracy

Intra-day accuracy and precision were computed through the analysis of RLMs and plasma matrix samples spiked with PNT and QC samples at various concentrations in one day. Additionally, inter-day measurements were done in a similar way on three successive days. For expressing accuracy and precision of the suggested methods, percentages error(% error) and percentages relative standard deviation (% RSD) were used respectively. Percentages RSD = (SD/Mean) ×100 and percentages error = [(average measured concentration—expected concentration) / expected concentration] ×100].

#### Assay recovery

The extraction recovery of PNT was evaluated by comparing the mean peak area of PNT in the QC samples with the mean peak area of PNT extracted from blank plasma and blank RLM that spiked with correspondent PNT reference solutions (n = 5).

#### Stability and dilution integrity

For assessing of PNT stability in RLMs and human plasma matrices, six replicates of QC samples were analyzed utilizing various storage conditions. Calculation of accuracy and precision values were carried out using freshly prepared RLMs and plasma calibration curves. PNT QC samples were stored at room temperature for 8 hours for further assessment of their bench top stability. Numerous freeze—thaw cycles (three cycle) were performed to estimate stability of PNT QC samples after freezing these samples at -80°C and then thawing them at room temperature. Additionally, assessment of PNT stability was done through assaying PNT spiked QC samples after one day of keeping them at 4°C and after one month of storing them at -20°C. Additionally, dilution integrity of PNT samples were done to confirm the integrity of the concentrated PNT samples that need e dilution step. PNT stock solution was prepared and spiked in plasma and RLMs to attain a concentration of 1.8 times of the upper concentration in the calibration range then two or four fold dilution were done. Three replicates of each dilution were evaluated and the integrity of the PNT samples was maintained if % nominal lied in ±15% of nominal values and % CVs ≤ 15%.

### Metabolic stability of PNT

The current metabolic stability study is conducted to trace any observable decrease in the concentration of PNT when incubated with RLMs matrix using LC-MS/MS method. Incubations of 1 μM PNT were carried out (in triplicates) with 1 mgmL^-1^ of microsomal proteins in addition to 1mM NADPH in phosphate buffer (pH 7.4) containing 3.3 mM MgCl_2_ in a final volume of 1 mL. The incubation is started by the addition of NADPH and terminated with 2mL of ACN at 0, 2.5, 5, 10, 15, 20, 40 and 50 min. Precipitated proteins were removed by centrifugation at 14000 rpm for 12 min at 4°C. Supernatants was removed and filtered through 0.22 μm syringe filter. IS (75 μL) was added to 1mL of the filtered supernatant in addition to 0.5 mL of mobile phase (to make the final conc. of PNT lied in the linearity range). Five μL of this solution was injected into the LC-MS/MS system. Concentrations of PNT in RLMs matrix were calculated from the regression equation of freshly constructed calibration curve using peak area ratios of PNT and IS.

## Results and Discussion

### Chromatographic separation and mass spectrometry

**Fig 2:**
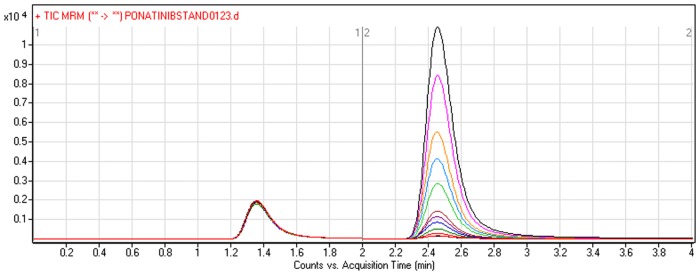
TIC chromatogram of MRM of PNT (5–400 ngmL^-1^) and IS (100 ngmL^-1^).

Several assessments were done to obtain the highest stabilized mass response through optimizing all analytical conditions so as to improve the sensitivity and resolution. The *p*H of the mobile phase (its aqueous portion) can assist the ionization of the analytes and also can adjust its peak shape. Different mobile phase compositions were examined where optimum isocratic mobile phase composition was acetonitrile-water (1:1) containing 10mM ammonium formate and pH ~ 4.1 (adjusted with formic acid). PNT and vandetanib (IS) retention times were 2.5 and 1.3 min, respectively, under the optimum chromatographic conditions. A run time of 4 min. was capable for good separation of the two analytes (PNT and IS) and no carryover was observed in blank matrix sample (RLMs or plasma alone) or PNT-free standard sample (blank + internal standard). Fig 2 showed the chromatogram of calibration standards of PNT and IS curve.

**Fig 3:**
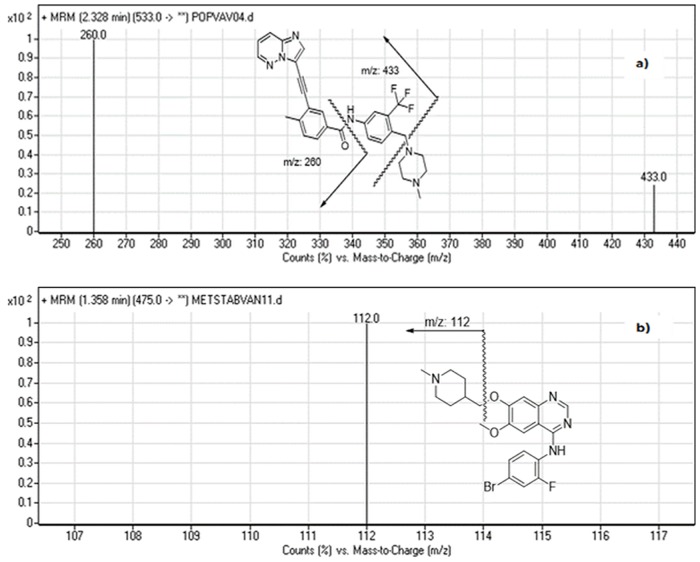
MRM mass spectra and the expected fragmentation pathway of (a) PNT and (b) vandetanib (IS).

In the same way, the parameters of mass spectrometry were adjusted to improve the ionization effectiveness of the parent ion as well as the major product ions of PNT and IS. MRM mode was adopted in this study to eliminate possible interference signals and enhance the sensitivity of the method. The mass spectra of the analytes principally comprised of one ion at *m/z* 533 for PNT and at *m/z* 475 for vandetanib (IS). Product ion scan for PNT positive ion at *m/z* 533 gave major ions of [M+H]^+^ at *m/z* 433 and 260. Correspondingly, IS ion at *m/z* 475 gave one fragment ion of [M+H]^+^ at *m/z* 112. These ions were selected for MRM mode scanning of PNT and vandetanib (IS) in the quantification method (Fig 3).

### Method validation

#### Specificity

The developed method was specific as no interference from components of RLMs or plasma matrices at the retention time of PNT and/or IS. The mass detector showed no carry over effect from samples. PNT and IS were well resoluted under the optimized chromatographic conditions with retention times were 2.5 and 1.3 minutes, respectively.

#### Linearity and sensitivity

The suggested method was sensitive and reliable for assaying PNT in human plasma or metabolic stability method. The linear regression analysis for the results was carried out using the least-square method. The results of the calibration curve showed linearity in the range of 5–400 ngmL^-1^, with a correlation coefficient (r^2^) ≥ 0.9998 in the two matrices. The calibration curves of PNT in plasma and RLMs have the regression equations of y = 1.517x + 0.3611 and y = 1.537x + 0.1986 respectively. The *LOD* and *LOQ* were found to be 1.53 and 4.66 ngmL^-1^ in plasma, 1.38 and 4.19 ngmL^-1^ in RLMS. The high r^2^ value was indicative for the good linearity, and the low values of standard deviations of the intercept and the slope were indicative for the significant validity of the calibration points used for constructing the calibration curve.

The relative standard deviation values of each concentration point (six replicates) did not exceed 2.93%. Calibration and quality control samples of PNT in plasma and RLMs matrices (eleven points) were back-calculated to confirm best performance of the suggested procedure. The precision and accuracy for PNT in plasma samples were found in the range of 0.82 to 2.93% and -2.45 to 2.17%, respectively. On the other hand, precision and accuracy in RLMs were ranged from 0.36 to 2.18% and -1.95 to 2.70%, respectively (Table 1). The mean recoveries % of PNT were 99.49±1.53% and 100.03±1.52% in plasma and RLMs, respectively. These results reflect the superiority of the current method over the reported spectrofluorometric method [8] (where PNT recovery % in plasma was around 85%).

**Table 1:** Data of back-calculated PNT concentration of the calibration standards from RLMs and plasma matrices.

Nominal Concentration(ngmL^−1^)	Plasma			RLMs		
	Mean^a^	Precision (RSD %)	Accuracy (RE %)	Mean^a^	Precision (RSD %)	Accuracy (RE %)
5	4.94±0.09	1.75	-1.14	4.97±0.08	1.53	-0.60
10	10.21±0.08	0.82	2.10	10.27±0.07	0.71	2.65
20	19.51±0.34	1.77	-2.45	19.61±0.16	0.80	-1.95
30	30.65±0.90	2.93	2.17	30.81±0.67	2.18	2.70
40	39.78±0.80	2.02	-0.55	39.99±0.54	1.35	-0.03
50	49.42±0.50	1.01	-1.16	49.69±0.23	0.46	-0.62
100	98.87±0.97	0.98	-1.13	99.41±0.51	0.52	-0.59
150	146.35±1.52	1.04	-2.43	147.14±0.22	0.15	-1.90
200	200.23±2.73	1.36	0.11	201.30±1.19	0.59	0.65
300	298.42±4.33	1.45	-0.53	300.01±1.93	0.64	0.003
400	398.01±3.77	0.95	-0.50	400.15±1.45	0.36	0.04

Average of six determinations

#### Precision and accuracy

The developed method was confirmed to be reproducible using intra- and inter-day precision and accuracy at PNT QC samples. Percentages error (% error) and percentages relative standard deviation (% RSD) were used for expressing accuracy and precision, respectively. The values for accuracy and precision lied into the suitable range following ICH guidelines [9, 10] as seen in Table 2.

**Table 2:** Intra-day and inter-day precision and accuracy of the proposed methods.

**In Human Plasma matrix**	**LQC (20 ngmL**^**-1**^**)**		**MQC (150 ngmL**^**-1**^**)**		**HQC (300 ngmL**^**-1**^**)**	
	Intra-day assay*	Inter-day assay**	**Intra-day assay**	**Inter-day assay**	**Intra-day assay**	**Inter-day assay**
**Mean**	19.70	19.89	147.95	147.72	299.21	299.48
**Standard deviation (SD)**	0.31	0.50	1.67	1.61	3.16	3.66
**Precision (%RSD)**	1.58	2.54	1.13	1.09	1.06	1.22
**Accuracy (%RE)**	-1.48	-0.55	-1.37	-1.52	-0.26	-0.17
**In RLMs matrix**	**LQC (20 ngmL**^**-1**^**)**		**MQC (150 ngmL**^**-1**^**)**		**HQC (300 ngmL**^**-1**^**)**	
	Intra-day assay*	Inter-day assay**	**Intra-day assay**	**Inter-day assay**	**Intra-day assay**	**Inter-day assay**
**Mean**	19.67	19.97	147.75	147.88	299.1	299.07
**Standard deviation (SD)**	0.29	0.46	1.59	1.86	3.08	2.90
**Precision (%RSD)**	1.49	2.31	1.07	1.26	1.03	0.97
**Accuracy (%RE)**	-1.65	-0.16	-1.05	-1.42	-0.3	-0.31

* Average of twelve determinations of day 1.

** Average of six determinations in three consecutive days.

#### Extraction recovery and matrix effects

Table 3 summarized the extraction recoveries of QC samples for determining the concentration of PNT in plasma and RLM matrices. The results were reliable, accurate and reproducible. To ensure the absence of matrix effect on the PNT analysis, six different batches of plasma ad RLMs matrices were extracted and spiked with 20 ng mL-1 of PNT (LQC) and vandetanib (IS) as set 1. Likewise, set 2 was prepared which comprised of six replicates of same concentrations of PNT and IS but dissolved in mobile phase. For evaluation of matrix effect, mean peak area ratio of set 1/set 2 × 100 was computed. The investigated plasma and RLMs matrices containing PNT exhibited 98.3 ± 1.54 and 99.17 ± 1.93, respectively. Mean RSD was 1.56–2.53 and 1.95–2.65% for plasma and RLM, respectively. Consequently, these results showed that plasma and RLMs matrices almost have no noticeable influence on the ionization of both PNT and vandetanib (IS).

**Table 3:** Recovery of QC samples for determining the concentration of PNT in plasma and RLMs matrix.

Nominal Concentration (ngmL^−1^)	Plasma			RLMs		
	20 ngmL^−1^	150 ngmL^−1^	300 ngmL^−1^	20 ngmL^−1^	150 ngmL^−1^	300 ngmL^−1^
**Mean**^**a**^	19.51	146.35	298.42	19.61	147.14	300.01
**Recovery (%)**	97.55	97.57	99.47	98.05	98.09	100.00
**Standard deviation (SD)**	0.34	1.52	4.33	0.16	0.22	1.93
**Precision (RSD %)**	1.77	1.04	1.45	0.80	0.15	0.64

#### Stability and dilution integrity

The results of stability studies and dilution integrity in plasma and RLMs were displayed in Tables 4 and 5 respectively. Quality control samples were used to perform stability studies. Stability was examined under different conditions. The deviation of the results from the mean value of the samples was less than 3.7% for plasma and 2.9% for RLMs. No noticeable loss of PNT was observed during the storage and handling of the QC samples at the tested conditions.

**Table 4:** PNT Stability data and dilution integrity in plasma matrix under different conditions.

Nominal Concentration(ng.mL^−1^)	Mean (ngmL^−1^)	Recovery %	Precision (CV %)	Accuracy (RE %)	
**Room Temp. for 8h**					
**20**	20.28	101.40	2.12		1.41
**150**	147.77	98.51	1.37		-1.49
**300**	298.54	99.51	0.67		-0.49
**Three freeze-thaw cycles**					
**20**	19.81	99.05	2.38		-0.94
**150**	147.86	98.57	1.09		-1.43
**300**	299.61	99.87	1.22		-0.13
**Stored at 4 o C for 24 h**					
**20**	20.29	101.45	2.17		1.46
**150**	147.87	98.58	1.57		-1.42
**300**	298.83	99.61	1.01		-0.39
**Stored at -20 o C for 30 days**					
**20**	19.62	98.10	1.65		-1.90
**150**	147.76	98.51	1.21		-1.49
**300**	298.93	99.64	0.96		-0.36
**Dilution integrity**					
**180**	176.49	98.05	1.63		-1.95
**360**	358.28	99.52	1.77		-0.48

**Table 5:** PNT Stability data and dilution integrity in RLMs matrix under different conditions.

Nominal Concentration(ng.mL^−1^)	Mean (ngmL^−1^)	Recovery %	Precision (CV %)	Accuracy (RE %)
**Room Temp. for 8h**				
**20**	19.86	99.30	1.48	-0.67
**150**	148.26	98.84	1.36	-1.16
**300**	298.25	99.42	0.96	-0.58
**Three freeze-thaw cycles**				
**20**	19.47	97.35	0.55	-2.63
**150**	147.25	98.17	0.63	-1.82
**300**	299.95	99.98	1.11	-0.02
**Stored at 4 o C for 24 h**				
**20**	20.34	101.70	2.07	1.68
**150**	146.98	97.99	1.19	-2.00
**300**	299.37	99.79	0.72	-0.21
**Stored at -20 o C for 30 days**				
**20**	20.20	101.00	2.07	0.97
**150**	148.99	99.33	1.48	-0.67
**300**	298.70	99.57	1.21	-0.43
**Dilution integrity**				
**180**	177.77	98.76	1.54	-1.24
**360**	360.85	100.24	0.50	0.24

For evaluation of dilution integrity of PNT samples, The mean recovery % and CV % for 1/2 and 1/4 dilution samples in plasma and RLMs were within 98–102% and <1.8%, respectively.

### Metabolic stability

**Fig 4:**
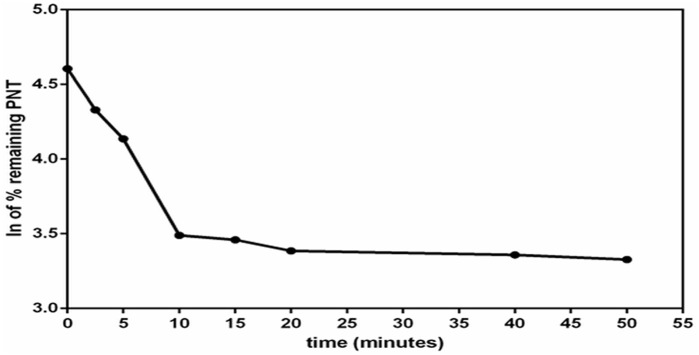
The metabolic stability profile of PNT after incubation with RLMs. Metabolic reaction was stopped at different time points.

After stopping the metabolic reaction at different time intervals, concentrations of PNT in RLMs matrix were calculated. These concentrations equal to the % remaining PNT with respect to zero time (which represents 100%). The ln of the % remaining PNT was drawn versus incubation time as displayed in Fig 4. Regression equation of the first linear part of the curve was used for calculation of *in vitro* t_1/2_ [12] (where *in vitro* t_1/2_ = ln2/slope). The slope was 0.1107 and hence *in vitro* t_1/2_ was found to be 6.26 min. Consequently, CL_int_ was calculated according to *in vitro* t_1/2_ method [13] and found to be 15.182 ± 0.477.

## Conclusions

A simple, sensitive and rapid LC-MS/MS method was developed and validated for assaying PNT in plasma and RLMs matrices. The current method showed a linearity range of 5–400 ngmL^-1^ with *LOD* less than 2 ngmL^-*1*^ in both matrices. Analysis time was so fast (lower than 4 min) with low solvent consumption. Assaying PNT samples in one day and/or in various days demonstrated the accuracy and precision of the proposed procedure. The developed method was applied successfully for the quantification of PNT in human plasma and RLMs with the evaluation of PNT metabolic stability in latter matrix. The metabolic stability was expressed in terms of *in vitro* t_1/2_ (6.26 min) and CL_in_ (15.182± 0.477).
